# New Perspectives on the Biogenesis of Viral Inclusion Bodies in Negative-Sense RNA Virus Infections

**DOI:** 10.3390/cells10061460

**Published:** 2021-06-10

**Authors:** Olga Dolnik, Gesche K. Gerresheim, Nadine Biedenkopf

**Affiliations:** Institute for Virology, Philipps-University Marburg, 35043 Marburg, Germany; dolnik@staff.uni-marburg.de (O.D.); gesche.gerresheim@staff.uni-marburg.de (G.K.G.)

**Keywords:** negative strand RNA viruses (NSV), viral inclusion bodies, biomolecular condensates, liquid-liquid phase separation (LLPS), viral replication, nucleoprotein, phosphoprotein

## Abstract

Infections by negative strand RNA viruses (NSVs) induce the formation of viral inclusion bodies (IBs) in the host cell that segregate viral as well as cellular proteins to enable efficient viral replication. The induction of those membrane-less viral compartments leads inevitably to structural remodeling of the cellular architecture. Recent studies suggested that viral IBs have properties of biomolecular condensates (or liquid organelles), as have previously been shown for other membrane-less cellular compartments like stress granules or P-bodies. Biomolecular condensates are highly dynamic structures formed by liquid-liquid phase separation (LLPS). Key drivers for LLPS in cells are multivalent protein:protein and protein:RNA interactions leading to specialized areas in the cell that recruit molecules with similar properties, while other non-similar molecules are excluded. These typical features of cellular biomolecular condensates are also a common characteristic in the biogenesis of viral inclusion bodies. Viral IBs are predominantly induced by the expression of the viral nucleoprotein (N, NP) and phosphoprotein (P); both are characterized by a special protein architecture containing multiple disordered regions and RNA-binding domains that contribute to different protein functions. P keeps N soluble after expression to allow a concerted binding of N to the viral RNA. This results in the encapsidation of the viral genome by N, while P acts additionally as a cofactor for the viral polymerase, enabling viral transcription and replication. Here, we will review the formation and function of those viral inclusion bodies upon infection with NSVs with respect to their nature as biomolecular condensates.

## 1. Introduction

As viruses are obligatory intracellular parasites, their replication cycle relies on essential processes in the infected host cell. Viruses thereby exploit and remodel the cellular architecture by inducing structural, functional, or biochemical changes to enable efficient viral replication.

**Figure 1:**
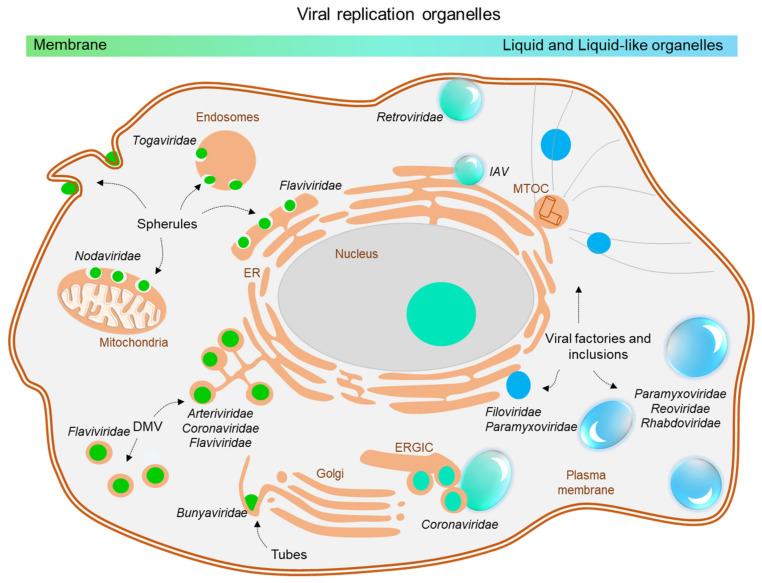
Overview on viral replication organelles in cells upon infection with different RNA viruses. Viral replication compartments associated with membranes are depicted in green, membrane-less compartments are indicated in blue. Those with liquid phase properties are depicted as droplets. DMV, double membrane vesicles. ER, endoplasmatic reticulum. IAV, Influenza A virus. MTOC, microtubule organizing center, ERGIC, endoplasmtic-reticulum–Golgi intermediate compartment.

During infection, many viruses induce the formation of distinct and specialized intracellular compartments that facilitate viral replication. Those specialized intracellular compartments are very heterogenous and designated as viral inclusions, inclusion bodies (IBs), viroplasms, virosomes, or viral factories and present a hallmark of viral infection [1,2]. Some of those compartments are connected directly to membranes, such as the endoplasmatic reticulum (ER) in Hepatitis C virus [3,4], dengue virus [5] or severe acute respiratory syndrome coronavirus (SARS CoV) 2 [6,7] infections, lysosomes in Semliki forest virus infection [8] or mitochondria (Flock House virus) [9]. These single- and double-membrane vesicles, convoluted membranes or tubular structures are a typical feature of infection by positive strand RNA viruses [10,11,12,13,14,15]. In contrast, viral inclusions during infections with many negative-sense RNA viruses are membrane-less but still localize in special cytoplasmic areas (summarized in Figure 1).

Recent investigations could demonstrate that many of those IBs share common properties with liquid organelles or biomolecular condensates. Those active, biochemically functional and membrane-less cellular compartments have become an emerging interest during the last decade. Biomolecular condensates display the properties of liquids and are highly dynamic and regulated structures in the cell involved in many different biological processes [16,17]. The underlying biophysical mechanism is, in most cases, regulated by liquid-liquid phase separation (LLPS), a mechanism similar to a water-in-oil-mixture [18,19]. The emerging investigations of these highly dynamic structures lead also to a paradigm change with respect to viral IB formation and function. Typical features of biomolecular condensates like dynamics, fusion activity, and reversibility are also characteristic for viral IB formation [19,20,21,22].

Here, we will review the current state of viral IB formation and function in infections with negative-sense RNA viruses, especially with respect to the emerging field of viral inclusions with properties of biomolecular condensates.

### 1.1. Biomolecular Condensates

In the last years, the physical properties of cellular molecules as a key factor for cellular organization gained more and more interest [16,17,22,23,24]. The mechanism and localization of biochemical processes have been for long years attributed solely to membrane-surrounded organelles. However, studies over the last decade have proven evidence of a cellular compartmentalization lacking a lipid boundary. Those membrane-less cellular structures are very heterogeneous in size and composition. Although they share similarities with the surrounding cytoplasm, they present separated, sometimes impenetrable cytoplasmic organelles that show a high dynamic plasticity and assemble/disassemble rapidly [16,25,26,27]. Owing to their biophysical properties that they share with liquids (like droplet fusion, surface tension, etc.), these biochemical functional compartments have been referred to as liquid (droplet) organelles or, more common in cellular biology, as biomolecular condensates [16,17,18,19,27,28,29,30]. Biomolecular condensates have been observed in different cells among eukaryots, bacteria, yeast, and archae [23,31,32,33,34]. They are ubiquitous observed across cellular compartments. In the cytoplasm, they are represented by stress granules, P-bodies, G-bodies [23,35,36,37,38] or in the nucleus, by nucleolus or Cajal bodies [39,40,41], for example. The main biophysical mechanism underlying the formation of biomolecular condensates is the LLPS, a mechanism similar to a water-in-oil mixture that leads to the separation of two (or even more) phases when left unperturbed [42,43]. The transition from soluble molecules to condensates (saturation concentration), liquid crystals, or aggregates is strongly regulated by thermodynamical factors like temperature, concentration, valency, and interaction strength between molecules [28,44,45,46,47]. The interphase of the different phases results intracellularly in the membrane-less boundary of biomolecular condensates that allow penetration by molecules with similar properties, while it excludes molecules with dissimilar features [20,22,43]. In cell biology, LLPS originates from protein:protein, protein:RNA, or RNA:RNA interactions that lead to the remodeling of a soluble phase into a condensated, dense phase. A key factor here is the multivalency of the molecules itself: multiple inter- and intramolecular connections that can lead to the formation of condensates with multiple interaction partners [28,29]. Certain properties of protein:protein interfaces have already been shown to drive protein phase separation: arginine-glycine-glycine/arginine-glycine (RGG/RG) motifs [48], charge-charge interactions and intrinsically disordered regions (IDRs) [49,50,51]. Interestingly, many IDR-containing proteins also have RNA-interaction interfaces. Under high concentration and molecular crowding, structured protein domains have also been described to drive LLPS [29,52]. Accordingly, posttranslational modifications such as phosphorylation or methylation also have a big impact on the formation of biomolecular condensates [53,54,55,56]. Expression of RNA and proteins, changes in their ratio, as well as RNA-scaffolded assembly of proteins all contribute to condensation and dynamics of LLPS in cells [35,57,58,59].

### 1.2. Viral Replication Cycle of Negative Strand RNA Viruses (NSV)

**Figure 2:**
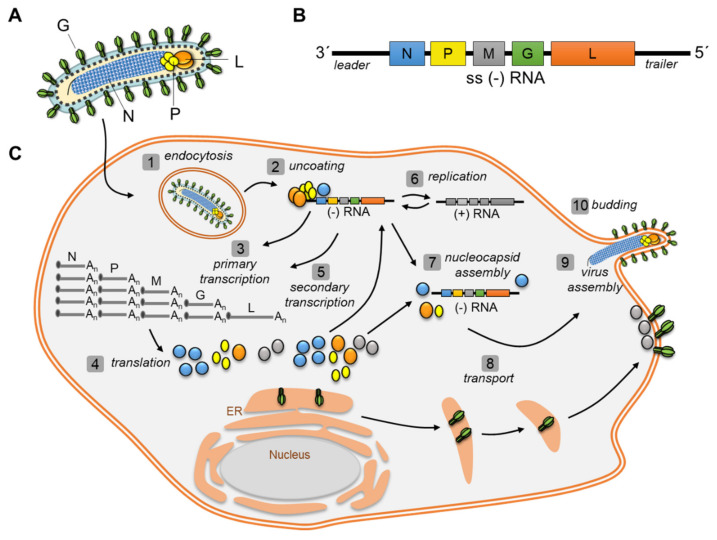
(**A**) Schematic diagram of a NSV particle. (**B**) General genome organization of a NSV. (**C**) Replication cycle of an NSV (based on a filoviral replication cycle). After entry into the cell **(1)** and release of the nucleocapsid into the cytoplasm **(2)**, primary viral transcription **(3)** is initiated by the integrated viral polymerase complex. Viral mRNAs are translated by the host translation machinery **(4)**. Synthesized viral proteins support new rounds of viral transcription **(5)**, replication **(6)** and nucleocapsid assembly **(7)**. Nucleocapsids are transported **(8)** to the cell periphery where they assemble to virions **(9)** and bud from the plasma membrane **(10)**.

The members of negative strand RNA viruses (NSV) comprise viruses that have a single-stranded, negative-sense RNA genome. They can be divided into virus families that have segmented genomes such as Orthomyxoviruses, Arenaviruses, and Bunyaviruses, or into non-segmented negative-sense RNA viruses (nsNSV, also termed *Mononegavirales*). The latter comprises several virus families (for example, *Paramyxoviridae, Bornaviridae, Rhabdoviridae, Pneumoviridae, Filoviridae*) with high relevance of individual representatives as human pathogens such as Measles virus (MeV), Nipah virus (NiV), Rabies virus (RABV), Respiratory Syncytial Virus (RSV), or Marburg and Ebola virus (MARV, EBOV) [60]. These viruses share a common architecture of their genomes. The RNA genome length varies between 12 (RSV, VSV) and 19 (filoviruses) kb in length and contains essential untranslated regions (UTRs) at their 3′- (*leader*) and 5′- (*trailer*) terminal ends important for viral transcription, replication, and encapsidation [61,62,63,64,65]. While the number of genes encoded by nsNSV varies among its families (from 5 to 10), the organization and relative position of the structural genes is highly conserved: 3‘- *leader*- Nucleoprotein N- Phosphoprotein P- Matrix protein (M)- Glycoprotein (G)- RNA-dependent RNA polymerase (L for large protein)- *trailer*-5′ (Figure 2A,B).

The RNA is tightly encapsidated in a non-covalently manner by the nucleoprotein (N, NP) that forms together with the other viral nucleocapsid or accessory proteins a helical Ribonucleoprotein complex (RNP) [61,66,67,68,69,70,71]. NSV are enveloped viruses that integrate their surface protein(s) (G, GP, Hemagglutinin H, or fusion protein F) into the host-derived membrane (Figure 2A). A layer of viral matrix protein(s) (M, VP40) represents the matrix that connects the membrane with the nucleocapsid. The replication cycle of nsNSV takes place in the cytoplasm of the host cell, with the exception of Bornaviruses that have a nuclear phase during their replication [72]. Entry of the virus is mediated by the attachment and binding of the surface protein to its receptor and fusion of the viral with the cellular membrane [73,74,75] (Figure 2C). Subsequently, the viral RNP is released into the cytoplasm of the cell. The RNP serves as template for viral RNA synthesis that starts (owing to the negative-sense genomic RNA) with primary viral transcription accomplished by the incorporated viral polymerase complex [76,77]. The RNA-dependent RNA polymerase (RdRp) L forms together with the phosphoprotein (P, VP35) the viral polymerase complex that enables mRNA synthesis of the viral genes [78,79]. Some representatives of the nsNSV encode additional viral nucleocapsid or accessory proteins that are essential viral transcription factors often regulated by phosphorylation (for example, VP30 for filoviruses or M2-1 for RSV) [77,80,81]. mRNA synthesis starts at the 3′-end of the genomic RNA and results in short, uncapped leader RNAs and 5′-capped, 3′-polyadenylated mRNAs [82,83]. Transcription of the monocistronic mRNAs is assumed to follow a start-stop mechanism regulated by highly conserved gene start and gene end sequences located in UTRs [78,84]. Polyadenylation of the viral mRNAs by the viral polymerase slows down transcription at the gene ends that may result in dissociation of the RdRp from the template. The result is a descending gradient of viral mRNAs from the first (N) to the last (L) gene, suggesting that the RdRp initiates transcription predominantly at the 3′-end of the viral genome and not from internal genes [76,82,85,86,87,88,89]. Following transcription, the cellular translation machinery translates the mRNAs into new viral proteins. Replication is carried out via the synthesis of a full-length antigenome in positive orientation that serves as a template for replication of the negative-sense genomic RNA. The switch from viral transcription to viral replication, when the RdRp ignores the transcription start and stop signals to synthesize the full-length antigenome, is not completely understood. It is suggested that the amount of newly synthesized N plays an important role to enable encapsidation of the nascent full-length antigenomic RNA during viral replication. N is synthesized as a monomer but starts to oligomerize quite rapidly and forms nucleocapsid-like structures, also with cellular RNA [90,91,92]. To prevent encapsidation of cellular RNA by N, N is kept soluble by the interaction with P [93]. The N^0^P complex, allows a concerted and regulated encapsidation of the viral RNA template [94]. However, different pools of polymerase complexes complemented by cellular and/or viral co-factors are also discussed to define either transcriptase or replicase activity of the RdRp [95]. Simultaneously with viral replication, genomic RNA serves again as template for further rounds of viral transcription accomplished by the newly synthesized polymerase complex components (secondary transcription).

Encapsidated genomic full-length RNA assembles together with the other nucleocapsid proteins to mature nucleocapsids that are condensed and transported along the cytoskeleton to the sites of viral budding at the cell periphery. The surface protein G co-localizes at the budding sites with the matrix protein M that drives the incorporation of the nucleocapsids into virions that are subsequently released from the plasma membrane [96,97,98].

### 1.3. Characteristics of Viral Inclusion Bodies (IBs)

New insights into the attributes of biomolecular condensate formation have also led to a reconsideration of viral IB formation in the virology field. High similarities of viral IB formation with biomolecular condensates driven by LLPS are obvious. Many viral IBs upon infection with nsNSVs have a high dynamic plasticity, they assemble/dissemble rapidly during infection, grow in size and appearance, and allow transport of exclusive molecules from in- or outwards. A major driving force in NSV IB formation is the expression of N and P proteins that are suggested as the basic scaffold in IB formation during infection. Two types of N-P interactions involving different interaction domains have been described: A monomeric N^0^P complex preventing association of N with cellular RNA, and nucleocapsid-associated P upon N oligomerization following its binding to genomic and antigenomic RNA [99]. All these steps involve multiple protein:protein and protein:RNA interactions that are mediated by highly conserved IDRs in both oligomeric proteins, P and N. All these attributes would contribute to the multivalent interactions underlying LLPS. Furthermore, several studies have demonstrated that de novo RNA synthesis occurs in viral IBs [100,101,102,103,104,105]. While the cellular protein synthesis itself is often shut down due to the viral infection, viral protein synthesis starts on a large scale. Simultaneously with an excess of viral protein expression, the viral RNA is subsequently replicated, encapsidated by the nucleoprotein and packaged with the nucleocapsid proteins.

All these different steps lead inevitably to strong changes in viral protein:protein interactions or protein:RNA interfaces, that might also contribute to LLPS in the viral IB and its surrounding. Understanding the biophysical mechanisms of viral IB biogenesis and regulation will also contribute to understanding the role and function of IBs for viral multiplication.

## 2. Viral Inclusions Formed upon Infection with Non-Segmented Negative Strand RNA Viruses (nsNSV)

**Figure 3:**
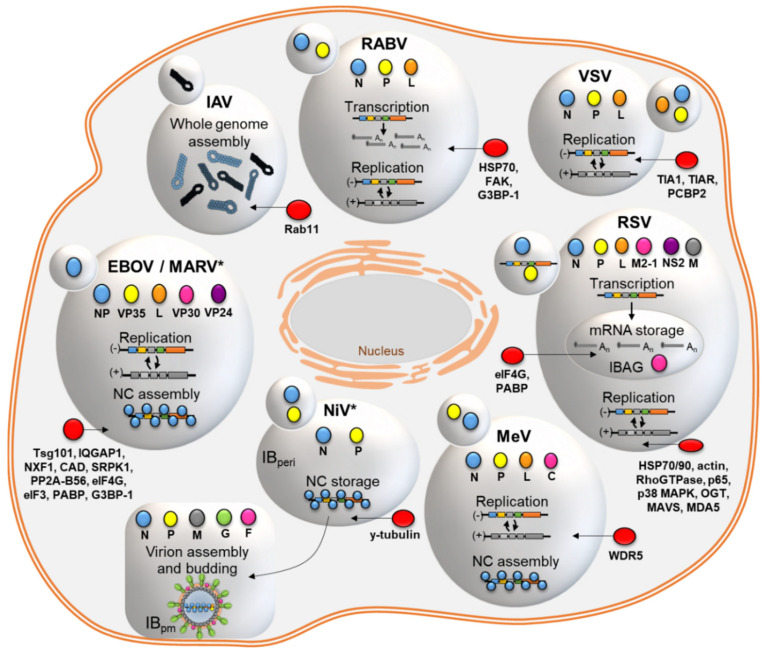
Summary of inclusion body (IB) formation upon infection with NSV. Small IBs indicate minimal required viral proteins for IB formation, while larger IBs represent mature IBs as biomolecular condensates formed by LLPS. * IB formation by LLPS suspected. Different steps of the viral life cycle taking place in IBs as indicated. In red cellular proteins that localize to IBs. RABV, Rabies virus [106]. VSV, vesicular stomatitis virus [103,107]. RSV, Respiratory syncytial virus [101,108]. MeV, Measles virus [109,110]. NiV, Nipah virus [111]. EBOV, Ebola virus [104,105]. MARV, Marburg virus [112,113]. IAV, Influenza A virus [114,115]. IBAG, IB associated granules. IBperi, perinuclear IB. IBpm, IB plasma membrane. NC, nucleocapsid. HSP70/90, heat shock protein 70/90. FAK, focal adhesion kinase. G3BP, Ras GTPase-activating protein-binding protein 1. TIA1, T-cell restricted intracellular antigen 1. TIAR, TIA1-related protein. PCBP2, Poly(RC) Binding Protein 2. p65, NF-κB subunit p65. p65 MAPK, p38 mitogen-activated protein kinase. OGT, O-linked N-acetylglucosamine transferase. MAVS, mitochondrial antiviral-signaling protein. MDA5, melanoma differentiation-associated protein 5. WDR5, WD repeat protein 5. Tsg101, tumor susceptibility gene 101. IQGAP1, Ras GTPase-activating-like protein 1. NXF1, Nuclear RNA export factor 1. CAD, carbamoyl-phosphate synthetase 2, aspar-tate transcarbamylase, and dihydroorotase. SRPK1, Serine-arginine protein kinase. PP2A-B56, protein phosphatase 2 B56 subunit. eIF4G, Eukaryotic translation initiation factor 4 G. eIF3, Eukaryotic initiation factor 3. PABP, Poly(A)-binding protein. Rab11, Ras-related protein Rab-11.

IB formation and changes in the phase separation due to viral infections might lead to the induction of essential subsequent steps of the viral life cycle like, viral RNA synthesis, encapsidation, assembly of nucleocapsid, and their transport to the cellular periphery. In the last years, there is significant new information about the replication of individual nsNSV in correlation with LLPS, which we will review in greater detail (summarized in Figure 3).

### 2.1. Rhabdoviridae: IBs of RABV and VSV

A prototype of IB formation upon infection with nsNSV are the Negri bodies that are formed in neurons upon infection with RABV [116]. These cytoplasmic IBs were named after their discoverer Aldechi Negri in 1903 and present a hallmark of rabies diagnosis in the central nervous system (CNS). Negri bodies have been described as places of viral transcription and replication [102]. Components of the viral replication machinery are hence localized in Negri bodies as well as the matrix protein M. Apart from that, cellular proteins like HSP70 and focal adhesion kinase (FAK) are recruited to those IBs [117,118,119]. Negri bodies were the first viral IBs that have been demonstrated to present organelles with liquid properties [106]. Using fluorescently labelled RABV together with live-cell imaging and FRAP (fluorescence recovery after photobleaching) technologies, the nature of Negri bodies as biomolecular condensates formed by LLPS was demonstrated. Negri bodies are spherical structures with fusion capacity; they show transit with vesicles and can be in reversible form once they encounter a physical barrier [106,116]. The highly dynamic formation of Negri bodies was shown by applying a hypertonic shock to the RABV-infected cells that resulted in the dis- and reappearance of Negri bodies in only 15 min. Interestingly, at later time points of infection, the shape of Negri bodies was changed and they were associated with membranes, most likely derived from the ER [106,116]. The minimal requirement for Negri body formation as a biomolecular condensate was the recombinant expression of N and P alone. However, the typical pinching off events seen from Negri bodies (most likely RNPs) were missing upon N-P expression, suggesting further viral or cellular factors that contribute to the final nature of Negri bodies. The key domains of P that mediate Negri body appearance in complex with N were narrowed down by mutational approaches to the dimerization domain, the amino-terminal part of its second intrinsically disordered domain (IDD2) as well as the C-terminus. IDDs in general have no stable three-dimensional structure, but instead show a high degree in flexibility that can result in binding to other proteins or RNA, as well as in post-translational modifications like phosphorylation [120]. In this regard, IDD1 and IDD2 of P are flanking a dimerization domain (DD) and, like the C-terminus, are phosphorylated. However, phosphorylation of P did not impact Negri body formation [106]. While it was previously shown that stress granules, also liquid organelles, are formed in close proximity to Negri bodies, fusion events or exchange of proteins between both could not be demonstrated, suggesting that both cellular compartments present separate phases within the cytoplasm [106,121].

VSV IBs appear first around 4 h post infection and are also the major site of VSV RNA synthesis. Primary viral transcription, however, is suggested to take place in the cytoplasm prior to IB formation [103]. VSV IBs were recently shown by live-cell imaging to present liquid organelles, whose formation is dependent on LLPS [107]. Disrupting the microtubule cytoskeleton with nocodazol resulted in round inclusions containing eGFP-P labeled VSV. Those IBs were able to fuse by random motion supporting the hypothesis of intrinsic surface tension of VSV IBs, a characteristic feature of LLPS. In contrast to other members of the nsNSV, besides the expression of P and N, IB formation additionally requires the expression of the VSV polymerase L [103,107]. This was tested by recombinant expression of the proteins and complementary by depleting viral protein expression in VSV-infected cells using puromycin, as global protein synthesis inhibitor, or protein-specific PPMOs (peptide-conjugated morpholino oligomers). In addition, the inhibition of M protein expression using a specific PPMO had no effect on the formation or properties of the IBs [103,107]. Using an inactive mutant of L, L G174A, revealed that IB formation is independent of viral RNA synthesis, suggesting that the nature of the protein:protein interaction is the driving force of VSV IB formation via LLPS.

### 2.2. Pneumoviridae: IBs of RSV

RSV IBs have been described as spherical cytoplasmic structures where viral transcription and replication occurs and to which all viral proteins of polymerase complex, N, P, L, M2-1 are recruited to enable viral RNA synthesis [101,122,123,124]. Besides the components of the viral polymerase complex, the nonstructural protein NS2 and the matrix protein M are recruited to RSV IBs [125,126]. RSV IBs also recruit cellular proteins involved in translation initiation, like the poly A binding protein PABP, translation initiation factor eIF4G [101], protein phosphatase 1 (for regulating RSV transcription mediated by M2-1 phosphorylation) [81] or heat shock proteins HSP90 and HSP70 [127,128]. Additionally, cellular proteins involved in nucleocapsid assembly and -transport like actin, actin-associated proteins and rhoGTPases like rac1, rhoA and cdc42 colocalize in IBs [129,130,131].

While genomic RNA could be detected in RSV IBs [124,132], a recent study confirmed additionally viral mRNA synthesis to be present in RSV IBs, independent of their size [101]. Live-cell imaging and pulse chase analyses with a fluorescently labelled recombinant RSV (M2-1 GFP fusion protein) underlined the dynamics of IB formation during the RSV replication cycle. A very intriguing finding of this study was the identification of a subcompartment inside the IBs by super-resolution microscopy, called IBAGs (for IB-associated granules), where newly synthesized viral mRNA accumulated together with the viral transcription activator M2-1, while N, P, L, and genomic RNA were excluded [101]. Formation of IBAGs was strongly dependent on viral RNA synthesis as their number increased during the viral replication cycle from 12 h p.i. on. Interestingly, while nascent viral mRNA and the cellular proteins PABP or eIF4G involved in translation initiation co-localized in IBAGs, other components of the cellular translation machinery, like the ribosomal subunit proteins S6 or L4, did not concentrate on IBs at all. As pulse-chase experiments could demonstrate that newly synthesized viral mRNA only transits through IBAGs, it is suggested that they might present rather transient mRNA storage sites but not sites of viral mRNA translation that most likely occurs in the cytoplasm. IBAGs share similarities with cellular stress granules that are formed by LLPS [133], although IBAGs do not contain typical stress granule proteins like G3BP or TIA-1. The minimal requirement of RSV IB formation is, like for RABV, expression of N and P alone [108,134]. The assembly of IBs was shown to be dependent on the RNA binding- and oligomerization capability of N and P, as N mutation towards a N^0^P complex was not sufficient to induce IB assembly in transfected cells [108]. With respect to P, it was demonstrated that the oligomerization domain as well as its C-terminus were essential for IB formation. FRAP experiments on expressed mCherry-tagged N and P proteins could demonstrate *in cellula* as well as in vitro that the formation of RSV IBs occurs by LLPS mediated by N- and P interactions [108].

### 2.3. Paramyxoviridae: IBs of MeV and NiV

In MeV-infected cells, all the components of the MeV polymerase complex N, P, and L, as well as C colocalize in cytoplasmic IBs where also viral RNA synthesis takes place [135,136,137,138,139]. However, IB formation is initiated by the recombinant expression of N and P alone, even in the absence of viral replication [109]. Extensive studies during the last years have been made to identify N and P domains that contribute to their interaction. Both proteins show a high plasticity with structured and disordered domains. N has been described to consist of a folded domain (N_CORE_) responsible for RNA binding, with two terminal arms followed by a highly flexible region called N_TAIL_ [140,141,142,143]. The tetrameric P contains a long intrinsically disordered (P_TAIL_) and a shorter disordered domain (P_LOOP_) [140,144,145]. P_LOOP_ is terminated by a small C-terminal three-helix bundle (XD) that has been shown to interact with RNA-associated N_TAIL_ and, for parainfluenzavirus 5 also with L [144,146]. In contrast, interaction in the N^0^P complex is mediated via the C-terminal domain of P [147].

Recent studies could demonstrate that MeV IBs represent biomolecular condensates formed by LLPS [109]. Characteristics of biomolecular condensates and LLPS, like a highly dynamic exchange between materials inside the IB with its surrounding, were observed by live-cell imaging upon MeV infection. IB formation was highly dynamic from small spherical structures to large inclusions. Interestingly, while smaller IBs were ubiquitously distributed in the cytoplasm, larger IBs appeared at the perinuclear region. By inhibition of dynein, a motor protein, the formation of perinuclear larger IBs was reduced suggesting that small cytoplasmic IBs transported dynein-dependent along microtubules to the cell nucleus to fuse towards larger IBs. Two further important assets of biomolecular condensates could be detected for MeV IB: the recruitment of cellular proteins (for example, eGFP- or mCherry-tagged WD-repeat protein (WDR) 5) [138], and recovery from photobleaching [109]. LLPS was initiated by the interaction of the C-terminal disordered region of N and P. N mutants that were unable to bind RNA could still form N- and P-mediated IBs. This suggests that RNP complexes, an often described driving force for LLPS [35,57,58,59], do not contribute to MeV IB formation [109], in contrast to RSV [108]. In vitro experiments using co-expressed N^0^P complexes and different mutants thereof confirmed that phase separation in vitro is also mediated by P and N interactions [110]. Interestingly interaction of P_XD_ and N_TAIL_ has been previously described to mediate the transport of the polymerase complex to the nucleocapsid prior to RNA synthesis [144,145,148,149,150]. Preventing the interaction between P and N by an N S491L mutation, a mutation that reduced viral transcription in cells [148,151], resulted in a complete abrogation of LLPS *in vitro*. The same was true upon mutation of P S86A and S151A indicating that P phosphorylation also contributes to phase separation. Interestingly, adding RNA to N-P droplets in vitro leads to the recruitment of RNA to droplets and triggered the encapsidation of RNA by N to nucleocapsid-like structures. The rate of encapsidation in those droplets measured by real-time NMR was enhanced when compared to the dilute phase [110]. These data confirmed the formation of nucleocapsid-like structures in these droplets and suggested a role of LLPS for the maturation of MeV nucleocapsids.

IB formation is also found upon infection by Mumps virus [152], Parainfluenzavirus 3 [153,154] and 5 [155] and NiV [111] and also initiated mainly by N and P expression. However, whether those IBs have properties of biomolecular condensates that contribute to efficient viral replication is so far not clear.

NiV, a highly pathogenic member of the *Paramyxoviridae,* differs from the other nsNSV by the induction of the formation of two distinct types of IB during infection. While one type is localized as spherical structures in the perinuclear region (IB_peri_), the second type characterized by a square shape is found at the plasma membrane (IB_pm_) [111]. Both types show not only different localization in the cell but also differ in their kinetics of formation and their content of proteins. While IB_peri_ are rapidly formed by N and P proteins upon transfection or early in infection, the matrix protein M is only found inside IB_pm_, suggesting that they present places of virion assembly and budding. However, fusion events could not be observed between IB_pm_, neither in transfected nor in infected cells suggesting a transport of nucleocapsids through the cell from one IB to another. Another very interesting finding was that IB_peri_ did not contain positive-sense RNA (mRNA or antigenomic RNA), suggesting that they represent no places of viral RNA synthesis, which is in contrast to many other nsNSV inclusions bodies. From this study, it is suggested that viral RNA synthesis takes place in a network of membrane-like reticular structures close to the ER [156], which is supported by the detection of nucleocapsids outside the IB_peri_. Whether LLPS and phase separation plays a role in the formation of IB_peri_ and IB_pm_ during NiV replication is so far not clear.

### 2.4. Filoviridae: IBs of MARV and EBOV

For Filoviruses, it is not clear whether IBs represent virus induced liquid-like compartments characterized by LLPS, as published so far for other NSV [106,107,110]. However, the current literature provides some evidence that this mechanism of compartmentalization, resulting in high functional dynamic and flexibility during the replication cycle, might be applied by filoviruses as well.

Live-cell imaging and time course studies showed that first small IBs appear in the perinuclear region of filovirus-infected cells [104,105] (and own unpublished data for MARV). The small IBs grow with time, they can fuse with each other, and in addition undergo fission events generating smaller IBs from bigger ones [104] (and own unpublished data for MARV). These observations were made using recombinant viruses expressing fluorophore-tagged nucleocapsid proteins like L-mCherry or VP30-GFP and might suggest LLPS processes during IB formation [104,112]. Earlier studies using single protein expression showed that the nucleoprotein NP alone induces the formation of IBs in transfected cells. All other nucleocapsid-associated proteins VP35 (a P analogue), viral nucleocapsid proteins VP30 and VP24, as well as L are diffusely distributed in the cytosol upon single expression and become IBs localized when co-expressed with NP [157,158,159]. The nucleocapsid proteins are important for the formation and structure of infectious nucleocapsids and possess in addition a wide range of functions in the filoviral replication cycle. VP35 is the co-factor of the viral polymerase L and inhibits IFN-signaling [160,161]. VP30 is a phosphorylation-dependent viral transcription factor necessary to initiate the formation of viral mRNAs [77]. VP24 is important for the formation and condensation of nucleocapsids and inhibits viral transcription and replication as well as innate immune response by interfering with interferon-mediated signaling [162,163,164,165].

It was recently shown that the C-terminal domain of EBOV NP is necessary for IB formation and that co-expression of VP35 can rescue IB formation upon expression of a C-terminal deleted NP [166]. This experiment suggests that IB formation and other functions of NP and VP35 involved in transcription and replication of viral RNA are separated processes, since RNA synthesis could not be rescued in this setting. Functional separation of different protein forms, for example, due to modifications like phosphorylation or different protein:protein complexes can occur by LLPS. Here, MARV and EBOV VP30 phosphorylation represent an example of how this modification changes protein:protein and protein:RNA interactions and influences its functions and localization in IB [77,167,168,169,170].

Viral RNA is the second important component detected in filovirus induced IBs [104,105,113]. Since early in infection, when primary transcription of viral mRNA takes place, IBs are not detectable, at later time points when protein translation starts and secondary mRNA transcription is initiated, IB formation colocalizes with *de novo* RNA synthesis and large IBs coincident with RNA replication [104,105]. Interestingly, IBs with different compositions of viral nucleocapsids proteins like L and VP35 were detected, suggesting the existence of different subsets of IBs with different functional properties [104]. The regulation of transcription and replication in filovirus IBs is still not understood and it has to be worked out if and how LLPS might favor one or the other process by formation of subcompartments, as shown for RSV IBAGs [101].

Ultrastructural analysis using electron microscopy identified IBs in filovirus-infected cells that contained nucleocapsids with different electron-densities [91,162,163,171,172]. Ectopic expression of nucleocapsid proteins revealed that thin-walled helices are formed in the presence of NP [171]. Thick-walled helices with high electron density can only be observed in the presence of NP, VP35, and VP24 [171]. The thick-walled helices are mainly located in the periphery of IBs, at the plasma membrane during viral budding and in extracellular virus particles [171]. It is presumed that the thin-walled helices represent RNPs, which serve as templates for the viral polymerase, and the thick-walled electron dense helices represent mature and transport-competent nucleocapsids in infected cells or nucleocapsid-like structures in transfected cells [162,171,173]. Therefore, a proper ratio of NP and VP35 in IBs seems to control the morphogenesis of nucleocapsids in EBOV-infected cells [174].

The formation of transport-competent nucleocapsids that have to be transported from the IBs to the budding sites seems to be highly dependent on VP24 functions in RNP condensation, which in turn blocks EBOV genome replication [162,165,175,176,177]. Ejection of transport-competent nucleocapsids from MARV and EBOV IBs correlates with high dynamics and the nature of described biomolecular condensates or liquid-like viral factories, which exchange material with the surrounding cytosol, as reviewed by Su and colleagues [112,178,179]. In addition, the transport of EBOV and MARV nucleocapsids from IBs to budding sites depends on actin polymerization, and the dynamic of IB assembly and disassembly is dependent on microtubules, representing a further characteristic described for liquid-like viral factories of other NSVs [104,112,178,180].

Filovirus IBs are not membrane-enclosed, as shown in many ultrastructural images; however, often located in close proximity to different cellular membrane compartments like ER, endosomal vesicles, and mitochondria [181,182,183]. Which and how host cell factors contribute to filovirus IB formation is not known. Several cellular proteins, like Tsg101, IQGAP1, NXF1, CAD and SRPK1, and others have been identified inside IBs, being important for different steps of the filovirus replication cycle [172,177,184,185,186,187]. Interestingly, it was published recently that ER contact sites regulate the dynamics of membrane-less organelles like P-bodies [188]. It is therefore also likely that filoviral IBs contact the different cellular compartments to enable material exchange, for example, viral and cellular proteins, and RNA, to favor different viral replication steps (transcription and translation, replication, assembly, condensation and transport of nucleocapsids). It remains to be analyzed if the required ATP provided by mitochondria and the necessary translation of viral and cellular proteins in close proximity to IBs might be covered and orchestrated by the mechanisms of liquid-to-solid transitions [22,189,190].

## 3. Viral Inclusions Formed upon Infection with Segmented Negative Strand RNA Viruses (sNSV)

IAV, a member of the *Orthomyxoviridae*, belongs to the segmented NSVs containing eight segments of RNPs inside the virion. The fact that most virions contain precisely eight segments of each type indicates that genome packaging in IAV infection is a highly regulated process [171,191,192]. It is suggested that the whole genome assembly of the eight segments takes place before transport to the plasma membrane, where the final assembly of the virion takes place [171,193,194]. In contrast to most other members of the NSVs, IAV replicate their genome in the nucleus. The eight viral RNP segments exit the nucleus and accumulate in IBs in a perinuclear region that enlarge in the course of infection [114,115,195]. Since IAV replication takes place in the nucleus, IBs are no sites of viral RNA synthesis. However, a recent study could demonstrate that IAV IB formation displays characteristics of liquid organelles or biomolecular condensates. Their formation in close proximity of the ER exit sites is spatially regulated, dependent on Rab11-GTPase and shows continuous cycling events of vesicles between the ER and the Golgi apparatus [115,196]. As expression of a single viral RNP could already initiate the formation of viral inclusions, viral IBs obviously occur before the assembly of whole genome RNP complexes. Sharing properties of biomolecular condensates, it is supposed that IAV IBs segregate viral RNPs from the cytosol to increase their concentration at hotspots that, in turn, facilitate the recruitment of other viral RNPs to allow assembly of whole IAV genome complexes [115]. Given the special feature of IAV genome reassortment, it is likely that IAV IB formation plays an important role in the assembly of newly reassorted IAV genomes.

The genus *Bunyavirales* contains viruses with either bi- or tripartite genomes containing the L, (M,) and S segments. In contrast to other bunyaviruses, the nonstructural proteins (NSs) of Severe fever with thrombocytopenia syndrome (SFTS) virus were able to form viral IBs upon transfection and infection, whichwas dependent on NSs self-interaction. It could be further demonstrated that those NSs-induced IBs contain the nucleoprotein and are places of viral RNA synthesis [197]. Interestingly, a colocalization of the SFTS IBs with lipid droplets was observed, and inhibition of lipid metabolism negatively affected SFTS replication.

For Bunyamwera virus, viroplasms have been described as tubular structures associated with the Golgi apparatus and the rough ER that are places of viral RNA synthesis and assembly [198]. For Junín virus (an Arenavirus), the nucleoprotein N was shown to induce the formation of discrete cytosolic IBs that may present viral transcription and replication centers. In contrast to most other nsNSV, those structures were associated with membranes and contained lipid metabolites [199].

However, whether these structures share biochemical properties with biomolecular condensates is so far not clear.

## 4. Role of NSV IBs in Antiviral Response

Given the spatial segregation of viral IB from the surrounding cytoplasm, it is also conceivable that IB formation may function as an additional viral escape strategy to avoid recognition by intracellular components of the antiviral defense machinery. Activation of pattern recognition receptors (PPRs) like RIG-1 and MDA5 recognizing cytosolic dsRNA leads to the activation of type 1 interferon and inflammatory responses combating viral infection [200,201,202]. A key determinant of antiviral activity are the viral phosphoproteins that are also key regulators for viral IB formation. The P proteins and their analogues have been described to block, for example, phosphorylation of the interferon regulatory factor 3 (IRF3) or IRF 7 [203,204,205,206,207,208], bind to dsRNA, and prevent RIG-I signaling or PKR activation [207,209,210].

Preventing activation of cell-intrinsic defense by IB formation could either be enabled by sterical exclusion or by concentrated sequestration of antiviral sensors avoiding activation of downstream pathways [211].

RSV antagonizes the innate immune response by sequestering cellular proteins involved in antiviral response activities into the IBs, such as NF-κB subunit p65, p38 mitogen-activated protein kinase (MAPK), O-linked N-acetylglucosamine transferase (OGT), mitochondrial antiviral-signaling protein (MAVS), and MDA5 [132,212,213]. Sequestration of MAPK p38 and OGT was suggested to suppress MK2 activity and formation of stress granules [213]. The cellular proteins were recruited to the IBs most likely via their interaction with N or P, suggesting an immune evasion strategy independent of the immunomodulatory RSV proteins NS1, NS2, or SH [212]. However, although the NF-κB subunit p65 was recruited to IBs, there was no co-localization with N and P suggesting that p65 localization might be regulated by other multivalent interactions within IBs [212].

Stress granules (SG), also liquid organelles with a role in antiviral activity [133,214] have been found in close proximity to RABV as well as VSV IBs [106,121,215]. While active fusion events between both biomolecular condensates could not be observed for RABV, the SG marker protein G3BP was found in some of the RABV IBs [106]. The function of G3BP localization in RABV IBs is unknown but may point towards the direction that LLPS may exclude antiviral proteins inside viral IBs to block antiviral downstream effectors [106]. For VSV IBs, in contrast, some SG proteins such as T-cell restricted intracellular antigen 1 (TIA1), TIA1-related protein (TIAR) or Poly(RC) Binding Protein 2 (PCBP2) co-localized to IBs [215]. The same is true for the EBOV IFN antagonist VP35 that can disrupt SG formation by sequestration of SG proteins into EBOV IBs (eIF4G, eIF3, PABP, and G3BP-1, but no TIA-1) to block innate immune responses [186,187].

For SFTS virus, a bunyavirus, it was demonstrated that sequestering of antiviral factors like IRF7, RIG-I, or STAT2 into viral IBs via the interaction with NSs leads to the suppression of IFN-alpha and -beta signaling pathways [216,217,218,219].

## 5. Conclusions

Over the last decade, the understanding of the intracellular architecture has changed tremendously by the discovery that intracellular membrane-less compartments represent liquid organelles or biomolecular condensates formed by LLPS. This also led to a paradigm change in the field of virology, especially with respect to the underlying mechanism of viral IB formation and maturation. For many NSVs, the liquid properties of IBs could be already demonstrated, with strong evidence that expression of N and P proteins are mostly the minimal requirement for IB formation (Figure 3). This could be attributed to their special protein architecture that includes multiple disordered regions and RNA-binding domains, hence multivalent interaction interfaces that contribute to LLPS. While RNA synthesis does take place in some of the NSV IBs, the structural role of RNA synthesis for LLPS formation and contribution to IB maturation is not fully understood, as well as the assembly of viral nucleocapsids in or from matured IBs. One may speculate that the molecular crowding of viral (and also cellular) proteins upon viral infection initiates the formation of IBs above a certain threshold, laying the foundation for the induction of further steps of the viral life cycle, possibly also driven by LLPS. In that regard, nucleocapsid assembly may be triggered as a result of the environmental changes induced by N and P expression and RNA synthesis.

Different cellular proteins interacting and co-localizing with viral proteins inside IBs have been identified so far. How they contribute to IB formation and LLPS is until now elusive. It is also feasible that many more cellular proteins might be recruited towards IBs due to their similar physicochemical properties, and maybe not all of them by their direct interaction with a viral protein. These interactions might be transient and require more live cell imaging and time laps studies and the use of super-resolution techniques. Research on the composition of IBs in cells will be an exciting field in the future, although challenging, since the liquid properties will make IBs purification difficult. The role of IBs in innate immunity, and how sequestration of cellular antiviral proteins into viral IBs may contribute actively to counteract antiviral activity will be also of great interest in the next years.

Future research on the biogenesis of viral IB formation and the underlying biophysical mechanism will help to understand how IBs promote viral replication, and may lay the foundation of the development of future antivirals, leading to the disassembly of viral IBs or that that may block viral RNA synthesis in place.

## Data Availability

Not applicable.

## Abbreviations

NSV	Negative strand virus
RNA	Ribonucleic acid
nsNSV	Non-segemented NSV
LLPS	Liquid-liquid phase separation
IBs	Inclusion bodies
ER	Endoplasmatic reticulum
IDR	Intrinsically disordered region
MeV	Measles virus
NiV	Nipah virus
RABV	Rabies virus
RSV	Respiratory Syncytial Virus
MARV	Marburg virus
EBOV	Ebola virus
IAV	Influenza A Virus
SFTS	Severe fever with thrombocytopenia syndrome Virus
N/NP	Nucleoprotein
P	Phosphoprotein
NS	Nonstructural protein
RNP	Ribonucleoprotein complex
PPMO	Peptide-conjugated morpholino oligomers
GFP	Green fluorescent protein
SG	Stress granules
